# Lectures of Prof. Agassiz before the Young Men's Association, of Buffalo

**Published:** 1854-03

**Authors:** 


*Lectures of Prof. Agassiz before the Young Men's Association, of Buffalo.*—This distinguished scholar and philosopher has, during the past month, delivered a course of five lectures on the relations of Zoology and Geology, to large and deeply interested audiences.

Of course, in so brief a space of time, he did not attempt to teach either of these sciences in detail. He ran rapidly through them, seizing their salient points, and presenting them in the clearest light; or, when detail was necessary to elucidate his subject, culling from his vast storehouse of facts precisely those illustrations most apposite to his purpose.

Prof. Agassiz has the most surprising facility as a lecturer, when we consider that he speaks in what is to him a foreign language. His accent is not, however, so marked as to interfere with intelligibility. But it is not so much his manner or language, which gives interest to his teachings, as the fact that he is not a mere man of detail; that immense as is his collection of facts, they are not isolated or dissevered from each other. He has them arranged with reference to each other, and is thus able to generalize from them. It is to this philosophical turn of mind that he owes his success.

In his last lecture he adduced those facts which go to prove the diversity of races, in contradistinction to the received doctrine of one common parentage. He gave rather the skeleton of an argument, than a full account of this subject, leaving much to be supplied by the minds of his hearers, and not distinctly avowing an opposition to the Mosaic record. It has occurred to us that a brief sketch of this lecture would be interesting to the medical reasoner, and for this reason we dot down such a report of it, as a somewhat leaky memory may furnish after a lapse of several days.

The different regions of the earth are peopled by different species and genera of animals. In the arctic regions we have animals which are found nowhere else; and this distinction extends, not only to variety of species, but to those great classes or genera which never merge into each other. Thus we have there no reptiles. In relation to fishes, they have their homes from which they never wander; specific regions of ocean, occupied by specific varieties of fishes. The fish of the Western Atlantic are entirely different from those on its European coast. Even those kinds which bear the same name, as the cod, and mackerel, are in reality very different fish. The same holds true of the fish of our streams. The trout occupies the mountain brook, and is never found in the large rivers in which the brook terminates.

So, too, of animals. Each zone of the earth has its own inhabitants. The lion, the tiger, the elephant, the rhinoceros, are denizens of Asia and Africa alone; and of particular portions of those continents. They are never found in Europe or America. Even in animals which bear the same name there is a specific difference. The bear, which inhabits all regions and countries, is subject to modifications in color and conformation peculiar to the region in which he dwells. He is the brown bear in Europe, the black bear to the east of the Rocky Mountains, the grizzly bear upon their western slope, and the white bear in the polar regions.

Those animals which have the greatest facilities for traveling, and seem to be adapted to different localities from the one which they inhabit, nevertheless do not migrate. They have a native country from which they do not wander, for which they seem to have been created, and set apart from the rest of the animal creation.

The modifications in color are also remarkable. The monkeys of Southern Asia have a yellow color, like their compatriots the Malays. In Africa he is much darker, while among the white races, in Europe, he does not exist at all in a state of nature, if we except those which are found on the European side of the Straits of Gibraltar.

The same differences mark the varieties of the human race. The Esquimaux does not merge into the Indian of our Northern States. He preserves his individuality, and does not emigrate. The Tartar races of Asia are confined to the same boundaries which, through all historical time, they have occupied.* The Caucasian race has ever occupied the home of civilization; it has ever progressed, not by amalgamation with other races, but by their destruction. The Negro, after centuries of enforced residence in the temperate zone, does not change his color. All the characteristics of the different races betoken a separate origin, as well as their continued residence in the same regions — a residence unchanged for ages, and not dependent, merely, on their ideas of comfort, but on a peculiar conformation which specially adapts them to their dwelling-place.

We have given thus succinctly, though imperfectly, a sketch of this most interesting lecture. While many will be found to dispute the doctrines it infers, all will give due credit to the honesty of purpose which dictated its sentiments. Prof. Agassiz spoke, not as a propagandist of strange doctrines, but as one who, having observed much, related those observations, and left all deductions, so far as possible, to the minds of his listeners. H.

* QUERY. Is not the Tartar invasion, and its usurpation of China, which has continued so many hundred years, form a partial exception to this rule ?

